# Performance of DNA methylation and blood-borne tumor indicators in detecting colorectal neoplasia and adenomas: a comparative study with the fecal occult blood test

**DOI:** 10.3389/fonc.2024.1373088

**Published:** 2024-10-31

**Authors:** Ming Chen, Ji Zhang, Bin Xu, Bilian Yao, Zhenzhen Wang, Ying Chen, Kaiyu Cai, Chenli Zhang

**Affiliations:** Department of General Practice, Ruijin Hospital, Shanghai Jiao Tong University School of Medicine, Shanghai, China

**Keywords:** FOBT, colorectal neoplasia, MSEPT9, mSDC2, adenoma

## Abstract

**Objectives:**

To evaluate the performance of stool methylated syndecan2 (mSDC2), methylated septin9 (mSEPT9), fecal occult blood test (FOBT), carcinoembryonic antigen (CEA), carbohydrate antigen 125 (CA125) and carbohydrate antigen 199 (CA199) in detecting colorectal neoplasia and adenomas.

**Methods:**

Blood-borne CEA, CA125, and CA199 levels were measured by electrochemiluminescence. The SDC2 methylation was detected by Methylation Detection Kit for Human SDC2 Gene (Real time PCR), and the SEPT9 methylation was detected by the Septin9 Gene Methylation Detection Kit based on PCR fluorescent probe assay. The colonoscopy combined with tissue biopsy pathology was used as a validation criterion for colorectal neoplasia.

**Results:**

In detecting colorectal neoplasia, the AUCs of mSDC2, FOBT and mSEPT9 were 0.935 (95% CI: 0.915-0.956, P<0.001), 0.824 (95% CI: 0.617-1.000, P<0.001) and 0.671 (95% CI: 0.511-0.831, P<0.001), respectively. The sensitivity of mSDC2, FOBT and mSEPT9 were 100.0%, 66.7% and 40.0%, respectively. But the AUC of CEA, CA125 and CA199 were not statistically significant for colorectal neoplasia (all P>0.05). The combined application of mSEPT9 and mSDC2 showed the best predictive performance (AUC: 0.956, 95% CI: 0.887~1.000). For adenomas, the AUC of FOBT was extremely low (AUC: 0.524, 95% CI: 0.502-0.545, P=0.004). The CEA, CA125, CA199, mSEPT9 and mSDC2 were not statistically significant in detecting adenomas (all P>0.05).

**Conclusions:**

For individual tests, FOBT and mSDC2 are relatively better indicators for detecting colorectal neoplasia compared to mSEPT9, CEA, CA125 and CA199. The combined form of mSEPT9 and mSDC2 to detect colorectal neoplasia has good predictive performance. However, none of these indicators demonstrated significant predictive power for detecting adenomas in our study.

## 1 Introduction

As a common gastrointestinal tumor, colorectal cancer is the third most common cancer in the world, causing more than 700,000 deaths annually and imposing a serious disease burden (1). As reported by the National Cancer Center, in China, the incidence of colorectal cancer continued to show an upward trend in 2016, with about 408,000 new cases, which made it the second most common cancer in the country (2). There is a significant gender gap in colorectal cancer incidence in China (3, 4). In particular, from 2000 to 2016, the average annual percentage increase in colorectal cancer incidence among Chinese men was twice that of women (2.4% vs. 1.2%) (4). To reduce the incidence rate, it is important to select appropriate indicators for colorectal neoplasia detection. Adenomas represent an early stage in the development of colorectal neoplasia, and increasing the detection rate of adenomas at an early stage has positive clinical significance.

Colonoscopy examination is currently the gold standard for the diagnosis of colorectal neoplasia due to its high sensitivity and specificity. However, this testing requires fasting and a long preparation time, which can reduce patient acceptance (5). In addition, as a costly and invasive test, colonoscopy examination has the potential to cause complications such as bowel perforation (6). The fecal occult blood test (FOBT) is a non-invasive, inexpensive screening test for colorectal neoplasia and has been suggested as a screening indicator (7, 8). Nonetheless, FOBT has low sensitivity and is susceptible to dietary intake, which can lead to false positives (9, 10).

DNA methylation is one of the important molecular markers of tumors and epigenetic alterations in cancer formation (11). SDC2 and SEPT9 methylation is considered to show potential value for screening colorectal neoplasia (8, 12–14). Whereas, the sensitivity of DNA methylation indicators for screening colorectal tumors varies significantly among different studies, and the conclusions are not yet uniform (8, 15–17).

Carcinoembryonic antigen (CEA), carbohydrate antigen 125 (CA125) and carbohydrate antigen 199 (CA199) are blood-borne indicators for the surveillance of gastrointestinal tumors, but their sensitivity is generally low (18–22). To date, there are limited comparative data on blood-borne tumor indicators and DNA methylation indicators for detecting colorectal neoplasia versus FOBT, which hampers clinical options.

To address the issue of the relative lack of comparative data between DNA methylation and blood tumor indicators, we validated colorectal tumors using colonoscopy examinations combined with pathological tissue biopsy as the gold standard. Additionally, the traditional FOBT was used as a reference to compare the clinical potential of various indicators in screening colorectal neoplasia and adenomas.

## 2 Methods

### 2.1 Study population

We included the general population undergoing medical check-ups from the Department of General Practice at our hospital, and population inclusion was performed through the principle of randomization. After inclusion and exclusion criteria, a total of 2096 people were finally included. The timeframe for the study was almost two years, from July 29, 2021 to April 24, 2023. The inclusion criteria were as follows: (1). Voluntarily undergoing a physical examination; (2). Willingness to undergo opportunistic screening, such as receiving tumor markers tests (CEA, CA125, and CA199), mSEPT9 tests, and colonoscopy tests;(3) Willingness to provide biological samples such as blood and faeces.

The exclusion criteria were as follows: (1). Failure to complete the colonoscopy test; (2). Previous history of gastrointestinal malignancy; (3). Chronic colitis such as inflammatory bowel disease and eosinophilic enteritis.

The study was approved by the Ethics Review Committee of Ruijin Hospital Affiliated to Shanghai Jiaotong University (IRB approval number: 2023-401).

### 2.2 Indicators detection and colonoscopy examination

The levels of blood-borne tumor indicators CEA, CA125, and CA199 were determined by electrochemiluminescence, and the cutoffs for positive biomarker values were 5 ng/mL, 24 U/mL, and 25 U/mL, respectively. Fecal Occult Blood Test (FOBT) is performed by physicians at Ruijin Hospital, Shanghai Jiao Tong University School of Medicine and follows the appropriate physical examination protocol.

Prior to performing the methylation assay, biological samples were processed for DNA extraction and bisulfite conversion and to ensure that the quality of the DNA met the analytical requirements.

The SDC2 methylation assay was performed using the Methylation Detection Kit for Human SDC2 Gene (Real time PCR) Colosafe^®^ (Creative Biosciences (Guangzhou) Co., Ltd.). A detected Ct value below 38 was defined as a positive result for SDC2 methylation (22). The Septin9 methylation assay was performed using the Septin9 Gene Methylation Detection Kit based on PCR fluorescent probe assay (Epi proColon 2.0 CE) (BioChain (Beijing)Science & Technology, Inc.). A detected Ct value below 41 was defined as a positive result for SEPT methylation (22).

The gold standard for diagnosis is colonoscopy combined with tissue biopsy pathology. Colonoscopy examination is performed by experienced and authoritative physicians at the hospital. All examinations were performed by 7 senior endoscopists, with the duration of endoscopic operations ranging from 8 to 28 years.

### 2.3 Statistical analysis

Statistical analyses were performed using the software R 4.3.2 (R Foundation for Statistical Computing, Vienna, Austria), and a two-tailed *P* < 0.05 was considered statistically significant. The Shapiro-Wilk test was used to determine whether the data conformed to a normal distribution. Median and interquartile range (IQR) were used for skewed continuous variables. Number and percentage (%) were showed for categorical variablesAfter fitting logistic regression, evaluation metrics such as area under the curve (AUC) of receiver operating characteristic curves (ROCs), sensitivity and specificity were further calculated to determine the performance of the indicators in detecting colorectal neoplasia and adenomas. We evaluated the predictive performance of combined indicators for detecting colorectal neoplasia using the gradient boosting machine (GBM) method. The predictive performance of indicators was evaluated using *R package pROC, reportROC, gbm and caret*.

## 3 Result

### 3.1 Baseline Characteristics of participants

Our study consisted of 2096 individuals, of whom 384 completed the FOBT test and 257 completed the mSDC2 test. As shown in **Table 1**, all subjects (2096 individuals) had completed CEA, CA125, CA199, and mSEPT9 tests. Additionally, in the total population (**Table 1**, 35.1% female, 21.3% ≥60 years), the polyp detection rate (PDR) and adenoma detection rate (ADR) were 50.7% (1,063 individuals) and 28.9% (605 individuals), respectively. Polyp pathology was observed to have the highest proportion of tubular adenomas with 562 cases (26.8%).

**Table 1:** Basic characteristics of participants.

Variables	CEA	CA125	CA199	FOBT	mSEPT9	mSDC2
Participants, N	2096	2096	2096	384	2096	257
Age, n (%)						
<60 years	1649 (78.7)	1649 (78.7)	1649 (78.7)	264 (68.8)	1649 (78.7)	187 (72.8)
≥60 years	447 (21.3)	447 (21.3)	447 (21.3)	120 (31.2)	447 (21.3)	70 (27.2)
Gender, n (%)						
Male	1361 (64.9)	1361 (64.9)	1361 (64.9)	246 (64.1)	1361 (64.9)	168 (65.4)
Female	735 (35.1)	735 (35.1)	735 (35.1)	138 (35.9)	735 (35.1)	89 (34.6)
Polyp pathology, n (%)						
Negative	974 (46.5)	974 (46.5)	974 (46.5)	156 (40.6)	974 (46.5)	103 (40.1)
Tubular adenoma	562 (26.8)	562 (26.8)	562 (26.8)	121 (31.5)	562 (26.8)	80 (31.1)
Melanosis coli	17 (0.8)	17 (0.8)	17 (0.8)	4 (1.0)	17 (0.8)	6 (2.3)
Serrated adenoma	8 (0.4)	8 (0.4)	8 (0.4)	2 (0.5)	8 (0.4)	0 (0.0)
Villous adenoma	25 (1.2)	25 (1.2)	25 (1.2)	6 (1.6)	25 (1.2)	4 (1.6)
Adenocarcinoma	10 (0.5)	10 (0.5)	10 (0.5)	6 (1.6)	10 (0.5)	2 (0.8)
Inflammatory polyp	37 (1.8)	37 (1.8)	37 (1.8)	2 (0.5)	37 (1.8)	3 (1.2)
Inflammation	42 (2.0)	42 (2.0)	42 (2.0)	9 (2.3)	42 (2.0)	5 (1.9)
Hyperplastic polyp	421 (20.1)	421 (20.1)	421 (20.1)	78 (20.3)	421 (20.1)	54 (21.0)
Polyp, n (%)	1063 (50.7)	1063 (50.7)	1063 (50.7)	215 (56.0)	1063 (50.7)	143 (55.6)
Adenoma, n (%)	605 (28.9)	605 (28.9)	605 (28.9)	135 (35.2)	605 (28.9)	86 (33.5)

FOBT, fecal occult blood test; mSEPT9, methylated Septin9; mSDC2, methylated SDC2; CEA, carcinoma equivalent antigen.

Among those who completed FOBT test (**Table 1**, 35.9% female and 31.2% aged ≥60 years), polyp pathology was highest for tubular adenomas (**Table 1**, 31.5%), with 121 cases. For those who completed mSDC2 examination (**Table 1**, 34.6% were female and 27.2% were aged ≥60 years), the highest proportion of tubular adenomas were observed.

### 3.2 Performance of indicators for detecting colorectal neoplasia

**Figure 1:**
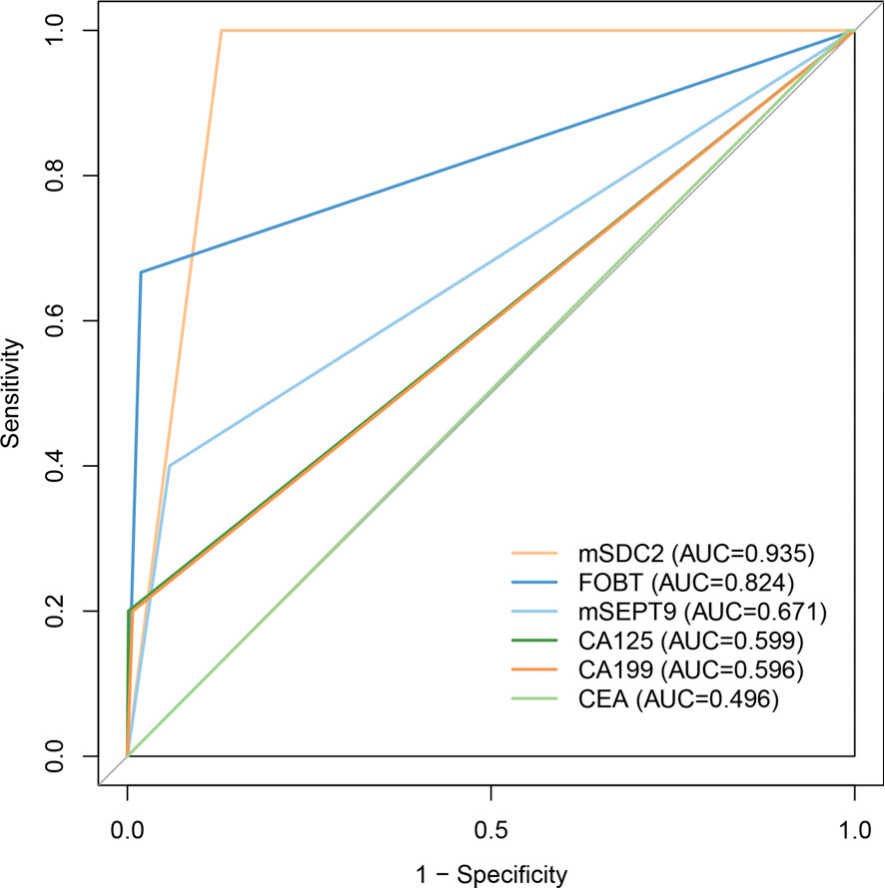
ROC curves of models for detecting colorectal neoplasia. Abbreviations were same as shown in **Table 2**.

For colorectal neoplasia, the AUCs for CEA, CA125 and CA199 were not statistically significant (**Table 2**, all P>0.05). And the AUCs for mSDC2, FOBT and mSEPT9 were 0.935 (**Table 2**, 95% CI: 0.915~0.956, P<0.001),0.824 (**Table 2**, 95% CI: 0.617~1.000, P<0.001) and 0.671 (**Table 2**, 95% CI: 0.511~0.831, P<0.001), respectively. Besides, mSDC2 (**Table 2**, Sensitivity: 100.0%) and FOBT (**Table 2**, Sensitivity: 66.7%) have relatively higher sensitivity compared to mSEPT9 (**Table 2**, Sensitivity: 40.0%). However, mSDC2 (12.9%) had a higher false positive rate than FOBT (1.9%) (**Figure 1**). Using combined indicators to detect colorectal neoplasia by the GBM method, we observed that the combined application of two methylation indicators, mSEPT9 and mSDC2, had the best predictive performance (**Table 3**, AUC: 0.956, 95% CI: 0.887~1.000). Interestingly, the combined form of mSEPT9, mSDC2 & FOBT does not show better predictive performance when one more indicator is added (**Table 3**, AUC: 0.955, 95% CI: 0.893~1.000).

**Table 2:** Performance of each indicator for detecting colorectal neoplasia.

Biomarkers	N	AUC (95% CI)	*P value*	Sensitivity %	Specificity %	PPV %	NPV %	PLR	NLR
CEA	2096	0.496 (0.494, 0.498)	0.385	1	0	–	0.995	1	–
CA 125	2096	0.599 (0.469, 0.730)	1	0.2	0.999	0.4	0.996	139.067	0.801
CA 199	2096	0.596 (0.466, 0.727)	1	0.2	0.993	0.118	0.996	27.813	0.806
FOBT	384	0.824 (0.617, 1.000)	<0.001	0.667	0.981	0.364	0.995	36	0.34
mSEPT9	2096	0.671 (0.511, 0.831)	<0.001	0.4	0.942	0.032	0.997	6.896	0.637
mSDC2	257	0.935 (0.915, 0.956)	<0.001	1	0.871	0.057	1	7.727	0

FOBT, fecal occult blood test; mSEPT9, methylated Septin9; mSDC2, methylated SDC2; CEA, carcinoma equivalent antigen; AUC, area under the curve; PPV, positive predictive value; NPV, negative predictive value; PLR, positive likelihood ratio; NLR, negative likelihood ratio.

Positive thresholds were defined as CEA (≤ 5ng/ml), CA125 (≤ 24ng/ml), CA199 (≤ 25ng/ml), mSEPT9 (Ct value ≤ 41), and SDC2 (Ct value ≤ 38).

**Table 3:** Performance of combined indicators for detecting colorectal neoplasia using the GBM method.

Performance	mSEPT9 + FOBT	mSDC2 + FOBT	mSEPT9 + mSDC2	mSEPT9 + mSDC2 + FOBT
N	384	215	257	215
AUC (95% CI)	0.818 (0.611, 1.000)	0.939 (0.917, 0.961)	0.956 (0.887, 1.000)	0.955 (0.893, 1.000)
Sensitivity %	0.667	1.000	1.000	1.000
Specificity %	0.937	0.878	0.871	0.878

GBM, gradient boosting machine; FOBT, fecal occult blood test; mSEPT9, methylated Septin9; mSDC2, methylated SDC2; AUC, area under the curve.

Positive thresholds were defined as mSEPT9 (Ct value ≤ 41) and SDC2 (Ct value ≤ 38).

### 3.3 Performance of indicators for detecting adenomas

For adenomas, the AUCs for CEA, CA125, CA199, mSEPT9 and mSDC2 were not statistically significant (**Table 4**, all P>0.05) except for FOBT (**Table 4**, AUC: 0.524, 95%CI: 0.502~0.545, P=0.004). However, the AUC of FOBT was less than 0.6 and the sensitivity was only 5.9%.

**Table 4:** Performance of each indicator for detecting adenomas.

Biomarkers	N	AUC (95% CI)	*P value*	Sensitivity %	Specificity %	PPV %	NPV %	PLR	NLR
CEA	2096	0.504 (0.499, 0.510)	0.977	0.015	0.994	0.5	0.713	2.464	0.991
CA 125	2096	0.503 (0.500, 0.506)	0.994	0.007	0.999	0.8	0.713	9.858	0.994
CA 199	2096	0.501 (0.497, 0.506)	0.722	0.01	0.993	0.353	0.712	1.344	0.997
FOBT	384	0.524 (0.502, 0.545)	0.004	0.059	0.988	0.727	0.66	4.919	0.952
mSEPT9	2096	0.512 (0.500, 0.524)	0.978	0.076	0.947	0.368	0.716	1.435	0.976
mSDC2	257	0.529 (0.482, 0.576)	0.897	0.174	0.883	0.429	0.68	1.491	0.935

Abbreviations as in **Table 2**.

Positive thresholds were defined as CEA (≤ 5ng/ml), CA125 (≤ 24ng/ml), CA199 (≤ 25ng/ml), mSEPT9 (Ct value ≤ 41), and SDC2 (Ct value ≤ 38).

## 4 Discussion

To complement the comparative data on DNA methylation and blood-borne tumor indicators for the detection of colorectal neoplasia and adenomas, we included six commonly used indicators in our study. Although some of these metrics have been studied in colorectal cancer screening, there is still a relative lack of comparative data on large sample sizes for multiple metrics. Our study provides data on DNA methylation indicators compared with FOBT, which can provide a reference for clinical decisions. The key finding of our study is that SDC2 methylation and FOBT each have relatively good performance in detecting colorectal neoplasia when used as a single independent indicator. In addition, the combined form of two methylation indicators, SDC2 and SEPT9, showed good performance in detecting colorectal neoplasia.

Although there have been more studies on DNA methylation in colorectal cancer screening, there are fewer current data comparing between multiple metrics, and there is the problem of small sample size. First, in China, the Septin9 gene methylation testing means in the clinical approval time is relatively short, and the high cost of testing, the current from the domestic large sample of the population of opportunistic screening research is relatively small. It is known that the current domestic study of Septin9 gene methylation test screening for colorectal cancer only included more than 400 participants (22). In contrast to this study, the present study had a much larger population. Second, the blood samples collected from the population in this study were all collected before taking laxatives for colonoscopy and were delivered in a ct-DNA test tube device, and the time between delivery and on-line testing was no more than 24 hours, which resulted in a smaller laboratory error and higher reliability than other studies.

Some of the currently available colorectal cancer methylation gene tests have shown high sensitivity and specificity in some reports in the literature (13, 16, 22), but in the opportunistic screening of the healthy population in this paper, they did not show a higher clinical value than FOBT for colorectal malignancies or for advanced adenomas. First, the evolution of colorectal malignancies is relatively long (23), the timing of methylation of oncogenes and tumor suppressors in tumor tissue has not been clearly defined, and the abundance of free DNA in peripheral blood varies widely among individuals. Second, methylation detection of intestinal oncogenes can be detected earlier in tumor tissue, while the chance of detection after release into peripheral blood is greatly reduced, reducing their sensitivity for opportunistic screening in the general population (13, 24).

The sensitivity of SEPT9 screening for colorectal cancer in other studies ranged from 39.6% to 72% (15, 17, 25), whereas the sensitivity of SEPT9 screening for colorectal neoplasia in our study was 45.5%. Interestingly, SEPT9 methylation does not seem to perform well in colorectal cancer screening as mentioned in some studies (13, 16). This may be related to the source of the population, sample size and type of biological samples in different studies. Even though SEPT9 has been shown to distinguish tumors from normal mucosa. However, SEPT9 released from cancerous tissues has a significant delayed effect when released into the bloodstream, which may be one of the reasons for its low sensitivity. Moreover, other studies have observed that mSEPT9 in feces was more sensitive than mSEPT9 in plasma when screening for early-stage colorectal cancer (13). This could be due to the fact that mSEPT9 in stool tissue is more likely to originate directly from colorectal cancer tissue without being affected by the intestinal barrier, compared to mSEPT9 in plasma (13, 26).

In addition, epigenetic changes are reversible and dynamically regulated, not only by specific genes but also by environmental factors such as diet (27), alcohol consumption (28) and smoking (29). It should be noted that the present study was a single-center study, and a multicenter study would have included a higher representation of participants. Nevertheless, our multimetric screening study provides guidance for future clinical applications of methylation indicators, e.g., future comparisons of the screening performance of DNA methylation in biological samples from different sources (stool, blood, urine, etc.) are needed. In addition, the combined application of multiple DNA methylation indices may also provide a useful reference for clinical screening.

Notably, in our current study, the performance of mSDC2 and FOBT in screening colorectal neoplasia showed relatively better performance, which is similar to that in screening for colorectal cancer (8, 22, 30). DNA methylation plays an important role in the tumor formation stage (8, 31). With non-invasive and convenient characteristics, biomarkers of mSDC2 and SEPT9 have been widely used for early colon cancer screening (5, 16, 30, 32). Similar to other studies, the performance of SDC2 in screening for colorectal neoplasia showing a higher sensitivity than mSEPT9 (22, 33). On this basis, our study observed that plasma SEPT9 methylation exhibited poorer screening colorectal cancer performance than both SDC2 methylation.

To date, colonoscopy examination is the gold standard for screening for colorectal neoplasia. Nonetheless, patient acceptance is limited by its high cost and invasiveness. Annual guaiac-based FOBT is one of the key colorectal cancer screening programs recommended by the American Cancer Society’s (ACS) recently updated guidelines for adults (34). FOBT screening has a significant effect on reducing colorectal cancer mortality (35). And we observed that FOBT showed no worse performance than mSEPT9 in screening for colorectal malignancies in this study. Fecal occult blood testing can also be affected by intake of iron, vitamin C and animal offal, which can lead to false positive results. FOBT has been reported to have the limitation of low sensitivity for colorectal cancer screening (36, 37).

In our study, adenoma detection rates were low for all indicators, demonstrating a poor potential for adenoma detection. In other studies, the detection rate of plasma mSEPT9 for adenomas ranges from 14% to 23.3% (15, 22, 38, 39). The detection rate of adenomas by mSEPT9 in our study was 7.6%, which may be related to population heterogeneity. Similar to other studies, both CEA and CA199 had low detection rates of adenomas (22). Unsurprisingly, compared to early adenomas, malignant tumors are already at a later, more severe stage of tumor progression, where tumor-associated biomarkers are more likely to be detected. Thus, the search for sensitive biomarkers in the precancerous stage of malignant tumors, the adenomatous state, remains a pervasive and unresolved challenge.

There are still several limitations to our study. First, although data comparing DNA methylation with FOBT were provided in our study, the sample sizes for both metrics, SDC2 and FOBT, are still relatively small. To some extent, this will affect the statistical efficacy, so we still need to increase the sample size for further validation in the future. Secondly, the sample size of colorectal neoplasia identified by the gold standard of our study is relatively small, which still needs to be further verified in studies with larger sample sizes. Finally, in terms of study design, our inclusion of biomarkers related to DNA methylation for screening colorectal neoplasia was less comprehensive, such as genes like BCAT1, TFP12 and SFRP2 (18, 32, 40).

In conclusion, SDC2 methylation and FOBT are relatively better indicators for detecting colorectal neoplasia compared to mSEPT9, CEA, CA125 and CA199. The combined form of mSEPT9 and mSDC2 to detect colorectal neoplasia has good predictive performance. However, none of these indicators showed significant predictive power for the detection of adenomas in our study.

## Data Availability

The raw data supporting the conclusions of this article will be made available by the authors, without undue reservation.
